# Challenges and knowledge gaps in sex differences in cardio–kidney–metabolic syndrome across the lifespan

**DOI:** 10.1186/s13293-026-00878-w

**Published:** 2026-05-12

**Authors:** Aline M. A. de Souza, Alexandra Kautzky-Willer, Damian G. Romero, Licy L. Yanes Cardozo, Sofia B. Ahmed, Samar Rezq

**Affiliations:** 1 Department of Medicine, Division of Nephrology and Hypertension, Georgetown University, Washington, DC USA; 2 Division of Endocrinology and Metabolism, Department of Medicine III, Medical University of Vienna, Waehringer Guertel 18-20, Vienna, 1090 Austria; 3 Department of Pharmacology and Toxicology, University of Mississippi Medical Center, 2500 N. State Street, Jackson, MS 39216-4505 USA; 4 Mississippi Center of Excellence in Perinatal Research, University of Mississippi Medical Center, Jackson, MS USA; 5 Women’s Health Research Center, University of Mississippi Medical Center, Jackson, MS USA; 6 Cardiovascular Renal Research Center, University of Mississippi Medical Center, Jackson, MS USA; 7 Department of Medicine, University of Mississippi Medical Center, Jackson, MS USA; 8 Faculty of Medicine and Dentistry, University of Alberta, Edmonton, AB Canada; 9 Women and Children’s Health Research Institute, University of Alberta, Edmonton, AB Canada

**Keywords:** Adipose tissue, Brain vulnerability, Cardiovascular–Kidney–Metabolic syndrome, Estrogen and testosterone, Exogenous hormones, Pregnancy, Polycystic Ovary Syndrome, Sex differences

## Abstract

Cardiovascular–kidney–metabolic (CKM) syndrome represents a continuum of interrelated adiposity, insulin resistance, cardiovascular disease, kidney dysfunction, and metabolic disturbances that evolve across the lifespan. Emerging evidence demonstrates that both biological sex and sociocultural gender significantly shape CKM risk, progression, and clinical expression. CKM syndrome pathogenesis reflects complex multisystem interactions involving adipose tissue dysfunction, neurohormonal activation, inflammatory signaling, and vascular impairment, all of which exhibit important sex-specific patterns. This review examines CKM syndrome from a sex- and gender-informed perspective, highlighting how endogenous and exogenous sex hormones, reproductive transitions, pregnancy-related complications, and dietary exposures shape the long-term CKM syndrome risk. Particular attention is given to the roles of estrogen and testosterone in modulating adipose biology, vascular function, and metabolic regulation. Polycystic ovary syndrome is discussed as a model of androgen excess and multisystem metabolic vulnerability that accelerates CKM features. Finally, we address brain vulnerability within CKM syndrome, emphasizing shared inflammatory, vascular, and neuroendocrine mechanisms linking metabolic dysfunction to cognitive decline and neuropsychiatric disorders. Recognizing these interconnected and sex-specific influences is critical for advancing precision prevention and treatment strategies across the CKM syndrome spectrum.

**Graphical abstract:**

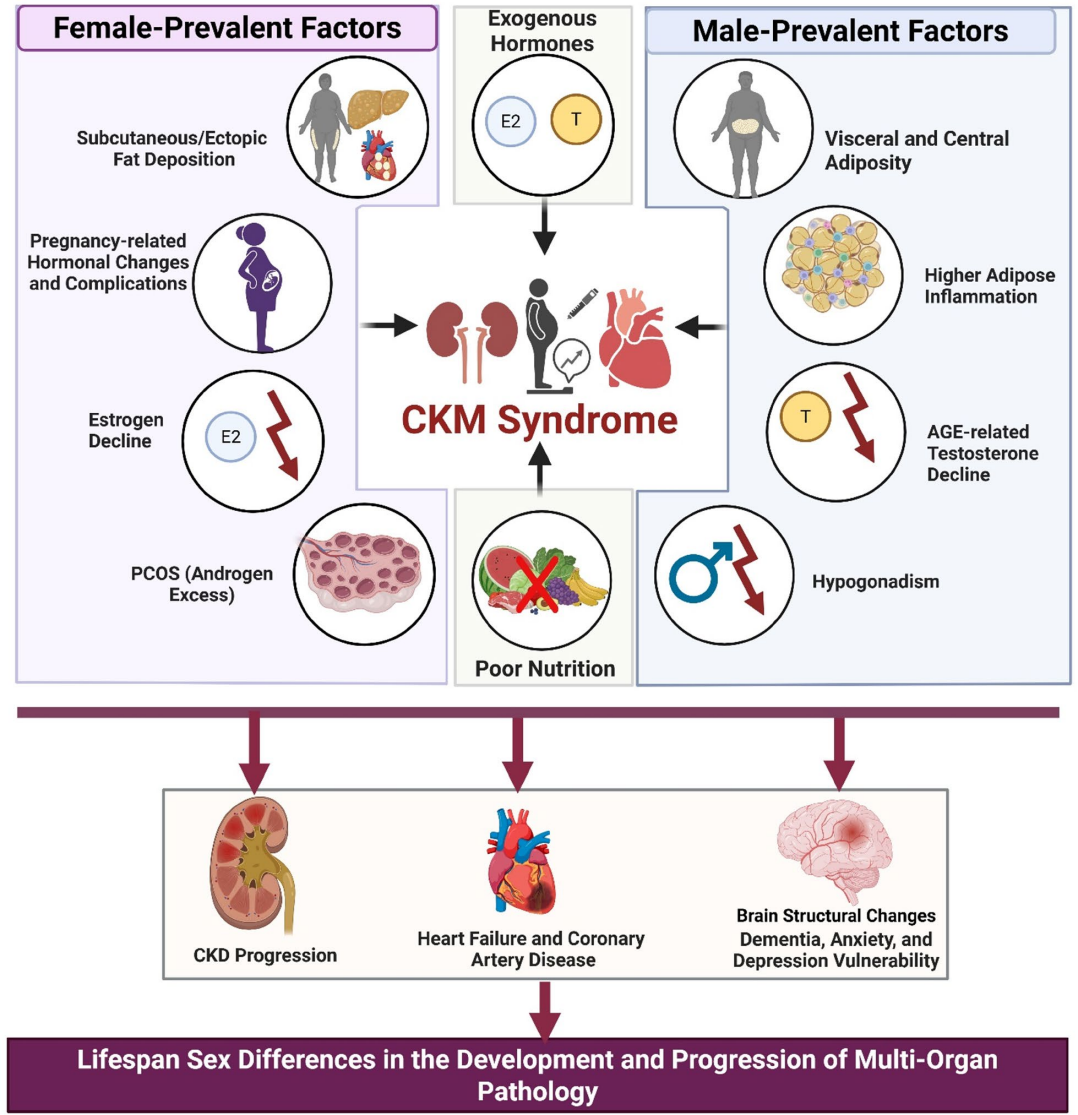

## Highlights

- Sex differences in adipose tissue distribution and hormone signaling drive divergent CKM syndrome trajectories. Visceral adiposity confers disproportionately greater cardiometabolic risk in women, whereas in men visceral fat accumulation and age-related testosterone decline promote insulin resistance, inflammation, and cardiovascular risk.
- Exogenous hormones are important determinants of CKM syndrome risk and outcomes, yet major gaps remain regarding their long-term cardiometabolic and renal effects, highlighting the need for longitudinal and mechanistic studies.
  Pregnancy represents both a sex-specific physiological adaptation and a cardiometabolic stress test that can unmask underlying CKM syndrome susceptibility.
- Polycystic ovary syndrome (PCOS) serves as a model of androgen excess and multisystem metabolic dysfunction, conferring early and persistent risk for CKM syndrome progression.
- Individual CKM syndrome components are linked to brain health in a sex-dependent manner across the lifespan, with women often showing greater vulnerability to adverse neurological outcomes.

## Introduction

The cardiovascular-kidney-metabolic (CKM) syndrome framework recognizes the pathophysiological interconnections among metabolic dysfunction, chronic kidney disease (CKD), and cardiovascular disease (CVD). The CKM staging system progresses from Stage 0 (no risk factors) through Stage 1 (excess/dysfunctional adiposity), Stage 2 (metabolic risk factors such as diabetes, hypertension, or moderate-to-high-risk CKD), Stage 3 (subclinical CVD or very-high-risk CKD), to Stage 4 (clinical CVD with metabolic/kidney disease) [1]. Each stage transition confers incrementally higher cardiovascular mortality risk, with approximately 90% of US adults affected by some stage of CKM syndrome [2]. Importantly, the 2023 American Heart Association Scientific Statement identifies an incomplete understanding of sex differences in CVD within CKM syndrome as a major knowledge gap [1].

CKM health and related outcomes come to the center of health [1]. The interplay among metabolic and renal risk factors and cardiovascular health markedly impact quality of life, morbidity, and premature mortality of all people. The prevalence of poor CKM health is increasing all over the world in both sexes, but disproportionally affects individuals with adverse economic, social, environmental, and psychosocial factors. In particular, in a cross-sectional analysis from the National Health and Nutrition Examination Survey (NHANES), unemployment, poverty, and food insecurity were found to be more likely related to advanced stages of CKM syndrome [3], factors that are indicators of health equity and relate to health outcomes. Moreover, living without a partner or living in a rented home increased the likelihood of advanced CKM stages only in middle age/old women [3]. Additionally, both sex and gender play an important role in pathophysiology, clinical presentation, and outcomes of CKM syndrome. However, there are still many research gaps, and up to now, action plans for evidence-based implementation in clinical care are missing. Addressing adverse social determinants of health next to biological sex differences may be crucial for the prevention and treatment of CKM syndrome in the future.

CKM syndrome can be initiated by excess and dysfunctional adipose tissue, in particular visceral and ectopic fat mass. Overall prevalence of obesity is higher in women than in men, but women more often feature healthy or preclinical obesity [4, 5]. However, body fat distribution, which is regulated by sex hormones, changes after menopause to a more adverse body shape, further increasing the risk of CKM syndrome in postmenopausal women. Obesity and metabolic risk factors, glucose intolerance or overt type 2 diabetes (T2D), hypertension, dyslipidaemia, and the related metainflammation increase the risk of CKM diseases as well as of CVD and heart failure. The overlap of comorbidities and inter-organ crosstalk also affects metabolic dysfunction-associated steatotic liver disease; the incidence also increases after menopause [4]. Interestingly, women showed a more pronounced increase in ectopic fat in the liver and heart with increasing body mass index (BMI) and deteriorating glucose metabolism compared to men of comparable age and BMI [6]. These changes could contribute to the higher relative risk of cardiometabolic disease in women with obesity and impaired glucose metabolism compared to their male counterparts. In NHANES, the prevalence of early CKM syndrome stages was found to be higher in women than men, whereas higher stages, including subclinical or clinical CVD were more often seen in men than women [7]. However, women featured higher hazard ratios for total mortality among all stages, including the end stage of clinical CVD in CKM syndrome. Moreover, the evaluation evidenced a continuous increase in the total prevalence of CKM syndrome during the last decades. Regarding kidney function, a strong relationship with sex hormones and age-related changes has been evidenced [8]; overall, women may be more protected by estrogen against apoptosis and cell senescence, and men appear more vulnerable to the progression of kidney disease with higher inflammatory response and higher risk of proteinuria related to testosterone levels. CKM health status also profoundly affects the reproductive health of women, with risk of pregnancy complications and early menopause [9].

The following sections address CKM syndrome from a sex- and gender-informed perspective, considering sex differences in adipose tissue and CKM risk progression within the cardio–renal–metabolic axis. Pregnancy, diet, and reproductive transitions are examined in relation to long-term risk, along with the roles of endogenous and exogenous hormones. CKM syndrome is further discussed in the context of Polycystic Ovary Syndrome (PCOS), a model of androgen excess and multi-organ metabolic vulnerability, and its implications for brain health and cognitive outcomes.

## Adipose tissue as a driver of CKM syndrome

Sex differences in adipose tissue biology represent a fundamental driver of divergent CKM trajectories. Premenopausal women preferentially accumulate fat in gluteofemoral and subcutaneous depots (the “pear” or gynoid distribution), whereas men accumulate more visceral and central adipose tissue (the “android” distribution) [10]. This sexually dimorphic fat partitioning has profound metabolic implications: visceral adipose tissue (VAT) is more strongly associated with cardiometabolic risk factors in women than men, with VAT conferring markedly greater odds ratios for diabetes (4.51 vs. 2.33 for BMI alone in women) and CVD death compared to anthropometric measures, while in men, BMI adequately captures VAT-associated risk [11]. In clinical practice, BMI is routinely assessed, whereas VAT quantification is not. This represents a particular disadvantage for women, as BMI alone may fail to capture cardiometabolic risk. Recently, the diagnostic criteria for clinical obesity require excess adiposity to be confirmed by either direct measurement of body fat or at least one anthropometric criterion (e.g., waist circumference, waist-to-hip ratio, or waist-to-height ratio) in addition to BMI [12]. Clinical practice should routinely incorporate waist circumference or waist-to-height ratio to provide a more comprehensive evaluation of metabolic risk than BMI alone, especially in women. Recent data from the Multi-Ethnic Study of Atherosclerosis demonstrate that within CKM Stage 3, subclinical atherosclerotic CVD predominates in men (70.5%), whereas subclinical heart failure phenotypes are more frequent in women (70.1%) [13].

##  Mechanistic underpinnings of adipose tissue in CKM syndrome: role of estrogen and testosterone

Estrogen regulates adipocyte proliferation and differentiation, favoring subcutaneous over visceral fat accumulation while suppressing appetite and increasing energy expenditure [14]. Experimental models strongly support these metabolic effects. Whole-body estrogen receptor-α (ERα) knockout mice develop increased total adiposity, preferential visceral fat accumulation, and features of the metabolic syndrome. Importantly, adipocyte-specific deletion of ERα (AdipoERα) results in increased adipose tissue fibrosis and inflammation, with these deleterious changes being more pronounced in males [15].

Testosterone exerts sexually dimorphic effects on adipose tissue that are central to understanding CKM syndrome pathophysiology. In men, testosterone deficiency is associated with increased visceral adiposity, reduced insulin sensitivity, and elevated cardiovascular risk, while testosterone replacement therapy decreases fat mass, increases lean mass, and enhances insulin sensitivity by upregulating insulin signaling genes (insulin receptor-β, IRS-1, AKT-2, GLUT4) in adipose tissue and suppressing inflammation [16]. Adipose tissue inflammation exhibits striking sexual dimorphism: men with obesity demonstrate profound macrophage accumulation, crown-like structures, and elevated proinflammatory cytokines, whereas women show less pronounced inflammatory infiltration despite reduced adiponectin [17]. In contrast, in women, androgen excess drives adipose dysfunction and metabolic deterioration; testosterone is positively correlated with adipose tissue insulin resistance (IR) in females but negatively correlated in males [18]. Mechanistically, androgen excess in females increases body weight, fat mass, food intake, serum leptin, adipose mitochondrial oxidative stress, IR, and adipocyte size, while decreasing serum adiponectin levels [19].

Collectively, these findings underscore adipose tissue as a central, sex-dependent driver of CKM syndrome progression. Reliance on conventional measures such as BMI may underestimate cardiometabolic risk in women, highlighting the need for sex-specific risk assessment strategies that better capture adipose distribution and its downstream consequences.

## Exogenous hormones and cardio-kidney-metabolic health and risk

Exogenous hormone therapies (menopausal hormone therapy [MHT] [20], hormonal contraception [21], gender-affirming hormone therapy [22], and testosterone replacement therapy [23]) are widely used across the lifespan and represent an important, yet under-recognized, potential determinant of CKM health. While these therapies exert significant effects on vascular function [24, 25], blood pressure regulation [26–30], metabolic homeostasis [28, 31–35], and kidney physiology [36–38], the magnitude, direction, and clinical significance of these effects vary substantially depending on hormone type, dose, formulation, route of administration, and timing relative to developmental and aging processes [39]. However, these factors are rarely captured in clinical datasets [40], limiting understanding of their impact on CKM outcomes. As a consequence, exogenous hormone exposure remains largely unaccounted for in CKM risk prediction models [41] and clinical practice guidelines [42]. This omission represents a critical limitation in current approaches to CKM risk assessment and management and represents a barrier to equitable and precise care.

Exogenous estrogen and testosterone therapies also alter body composition and muscle mass [32, 43–45], thereby affecting creatinine levels [46, 47] and potentially biasing creatinine-based estimates of glomerular filtration rate [48]. These physiological changes have direct implications for the diagnosis and staging of CKD, medication dosing, and assessment of cardiovascular risk. The *Kidney Disease: Improving Global Outcomes Women and Kidney Health Controversies* Conference emphasized that sex and gender influence diagnosis, risk assessment, prognosis, and treatment across kidney disease states, but also highlighted major evidence gaps, including hormonal exposures and life-stage transitions [9].

Regulatory actions underscore both evolving evidence and persistent uncertainty around the effects of exogenous hormone use on health outcomes. The United States Food and Drug Administration (FDA) recently requested labeling changes for menopausal hormone therapies, including modification of long-standing boxed warning language [49]. Similarly, following review of a multicenter randomized trial evaluating the cardiovascular safety of testosterone replacement therapy [50] and ambulatory blood pressure monitoring data, the FDA revised class-wide labeling for testosterone products by removing boxed warnings about cardiovascular risk while strengthening warnings related to increased blood pressure [51].

The largest knowledge gaps relate to both clinical outcomes and the mechanistic understanding of the effects of exogenous sex steroids. Longitudinal CKM outcomes, including incident hypertension, changes in body composition, IR, dyslipidemia, vascular dysfunction, CKD progression, and cardiovascular events, remain incompletely characterized across most exogenous hormone regimens, particularly among individuals with pre-existing CKM risk [52]. Bridging these uncertainties will require coordinated preclinical, translational, and longitudinal clinical studies that capture hormone regimen characteristics and link them to CKM phenotypes and outcomes.

## Pregnancy and CKM Health and Risk

Pregnancy represents one of the most distinguished sex-specific physiological and metabolic states influencing the CKM health. This period is a very delicate moment in a woman’s life because the woman’s body goes through innumerable physiological and metabolic changes throughout the course of a normal pregnancy. In early pregnancy, the hormones progesterone, estrogen, relaxin, and angiotensin system act in coordination to induce the blood volume expansion necessary for the fetus’s nutrition and development [53–55]. The plasma volume expansion happens because of systemic and renal vasorelaxation that activates the renin-angiotensin-aldosterone system, increasing renal sodium and water retention, increased renal blood flow, glomerular filtration rate, and increased cardiac output [53, 56, 57]. Because of those changes, pregnancy can be considered a stress test that can reveal unknown susceptibility to CKM syndrome.

The CKM syndrome or individual diseases such as hypertensive disorders of pregnancy, gestational diabetes, and impaired plasma volume expansion are common cardiometabolic complications with higher prevalence in low-income and middle-income countries [58]. Poverty, air pollution, educational and sociocultural barriers, and poor access to the health care system are some of the socioeconomic determinants of health during pregnancy [59]. Another variable that can affect pregnancy health, and is a current gap in the literature, is early-life nutrition. Food insecurity affects millions of people globally [60], and it can affect their health status later in life. Several studies have shown that undernutrition during childhood increases the risk of developing CKM syndrome during adulthood [61–64]. Yet females remain underrepresented in preclinical programming studies, and pregnancy as a second “hit” in this context is rarely investigated.

Conversely, when considering maternal health across the lifespan, there are several evidences that women who had preeclampsia, fetal growth restriction, and gestational diabetes have an increased risk to develop cardiovascular disease and CKM syndrome later in life [65–67]. These associations directly align with The Global Goals 3: Good Health and Well-Being [68], as maternal health is inseparable from long-term noncommunicable disease prevention. Despite all these associations, a clear mechanism remains unknown.

One major knowledge gap is whether pregnancy unmasks pre-existing neurohormonal hypersensitivity or induces long-term reprogramming of cardio-renal control systems. For example, exaggerated angiotensin II signaling, impaired endothelial barrier function, or reduced plasma volume expansion during pregnancy may contribute to persistent vascular stiffness, renal microvascular injury, and metabolic dysregulation postpartum. Yet longitudinal mechanistic studies tracking women from preconception through decades after delivery are scarce. Another important variable that also needs to be further explored, especially in basic science, is the age at conception and the risks of CKM syndrome. A comprehensive understanding of a woman’s prior nutritional history, current health status, and the age-related physiological adaptations required during pregnancy could enable a more individualized and preventive approach to reducing the incidence of CKM syndrome.

## PCOS and CKM syndrome

PCOS, affecting 5–18% of women of reproductive age, represents a critical intersection with CKM syndrome through its constellation of metabolic, cardiovascular, and emerging renal complications [69–74]. Women with PCOS demonstrate substantially elevated rates of CKM components, with metabolic syndrome prevalence 2- to 5-fold higher than BMI-matched controls and affecting 34–46% of U.S. Caucasian women with PCOS [75]. The syndrome is characterized by IR (often independent of obesity), dyslipidemia with lower high-density lipoprotein (HDL) and higher triglycerides and low-density lipoprotein (LDL) cholesterol, hypertension, and impaired glucose tolerance (30–35% of U.S. women) or T2D (8–10%) [70–72]. These metabolic derangements manifest across the lifespan, with adolescents showing unfavorable cardiometabolic biomarkers and postmenopausal women experiencing worsening IR, diabetes, and hypertension [74].

Emerging evidence now strongly demonstrates that women with PCOS experience increased cardiovascular disease events, not merely elevated risk factors. Recent large-scale meta-analyses and population-based cohort studies consistently show increased risk of myocardial infarction, stroke, and other major adverse cardiovascular events in women with PCOS [76–79]. Importantly, this increased CVD risk persists even in non-obese women without diabetes, suggesting PCOS confers risk beyond traditional metabolic factors [79]. These findings led the 2023 International Evidence-Based PCOS Guideline to recommend comprehensive CVD risk assessment in all women with PCOS [76]. Women with PCOS also demonstrate subclinical vascular disease, including endothelial dysfunction, increased carotid intima-media thickness, and elevated coronary artery calcium scores [69–72, 80]. While cardiovascular morbidity is clearly elevated, the relationship with cardiovascular mortality remains less certain and requires further investigation [76].

The kidney component of CKM syndrome in PCOS represents an emerging but understudied area. Recent evidence suggests potential associations between PCOS and CKD, with proposed mechanisms including chronic low-grade inflammation, hormonal dysregulation, and lipid metabolism disturbances [81]. Mendelian randomization analysis identified a positive causal association between PCOS and CKD (OR 1.180, 95% CI 1.038–1.342), with relationships to serological markers including fibroblast growth factor 23, creatinine, and cystatin C [82]. However, conflicting data exist, with one long-term population-based cohort study finding comparable CKD risk between PCOS patients and controls [83]. Major knowledge gaps include the pathological mechanisms, clinical manifestations, and progression of renal dysfunction in PCOS, as well as whether specific PCOS phenotypes confer differential renal risk.

Critical research priorities to fulfill our knowledge gap of the association of CKM syndrome and PCOS include: (1) prospective studies with standardized PCOS diagnostic criteria and comprehensive CKM phenotyping across the lifespan; (2) investigation of whether PCOS phenotypes (obese vs. lean, metabolic vs. reproductive, hyperandrogenemic vs. normoandrogenemic) demonstrate differential CKM risk trajectories; (3) elucidation of mechanisms linking PCOS to both cardiovascular events and renal dysfunction; (4) long-term cardiovascular outcome trials evaluating whether early pharmacological interventions reduce CVD event rates in PCOS; (5) assessment of ethnic and racial variations in PCOS-associated CKM risk, given evidence of phenotypic differences across populations. Understanding these relationships is essential for developing targeted prevention and treatment strategies for this high-risk population of women.

## Brain vulnerability in CKM Syndrome

Recently formalized by the American Heart Association, CKM syndrome describes the interaction of adiposity, IR, T2D, hypertension, dyslipidemia, and kidney dysfunction in accelerating vascular damage and multiorgan injury [84]. Each component independently increases the risk of neurological disease. Sex and gender further modify this risk, as chromosomal, hormonal, and sociocultural factors shape brain structure, function, and vulnerability across the lifespan [85]. Although direct evidence linking CKM syndrome to brain health is limited, substantial evidence connects its individual components to adverse neurological outcomes.

Among these components, metabolic dysfunction has been consistently associated with adverse neurological outcomes. Large epidemiological studies show that diabetes and metabolic syndrome significantly increase the risk of Alzheimer’s disease (AD), all-cause dementia, and vascular dementia [86, 87]. Adverse metabolic profile (hyperglycemia and dyslipidemia) is associated with the risk of depression, anxiety, and stress-related disorders, highlighting a link between metabolic dysfunction and psychiatric illness [88]. The underlying mechanisms likely involve shared vascular, inflammatory, and insulin-mediated pathways. IR and chronic inflammation may impair neuronal insulin signaling and promote neuroinflammation, contributing to AD-related pathology and cognitive impairment [89, 90]. Obesity and IR are additionally linked to compromised hippocampal structural integrity, with female sex recognized as an independent risk factor for this vulnerability [91]. Notably, while T2D increases the risk of dementia in both sexes, women were showing a higher risk for vascular dementia compared to men [92]. In the Amsterdam Ageing Cohort (mean age 79 ± 6.6 years), diabetes was associated with worse executive function, processing speed, and language performance, along with a higher incidence of cerebral lacunes and brain atrophy only in women [93]. In women, the sharp drop in estrogen during menopause, typically in midlife, is linked to increased central adiposity and adverse metabolic changes [94]. Other factors such as reproductive history, menopause timing, and lifetime estrogen exposure also influence brain vulnerability in women, pointing to sex hormones as key modulators of metabolic impacts on the brain [95]. In contrast, men experience a more gradual decline in testosterone beginning in midlife, which is also associated with increased visceral adiposity and higher risk of cognitive impairment [96–98]. These hormonal and metabolic changes may also intersect with other CKM syndrome components, including CKD, further contributing to brain vulnerability.

CKD could be both associated with and predictive of impaired mental health [99, 100]. Impaired renal function is linked to greater risk of cognitive decline, dementia, and psychiatric symptoms, likely through shared mechanisms such as vascular injury, inflammation, blood–brain barrier disruption, and toxin accumulation [101]. In CKD, cognitive impairment arises from vascular factors such as inflammation, hypercoagulability, and endothelial dysfunction, as well as nonvascular contributors including anemia, medication burden, and sleep disturbances. Metabolic abnormalities and uremic toxin accumulation further promote neurotoxicity, accelerating cognitive decline [102]. Notably, these kidney–brain interactions differ by sex, with men and women showing distinct patterns of cognitive effects and linked cardiovascular contributions [103]. Women with CKD show higher rates of depression and symptom burden [104, 105], whereas men often experience faster progression to end-stage kidney disease [106]. These sex differences may partly reflect the opposing vascular and inflammatory effects of estrogen and testosterone on the cerebral circulation, with age-related shifts in hormonal balance further modifying cerebrovascular and cognitive vulnerability across the lifespan [107].

Given its multisystem nature, CKM syndrome likely involves complex heart–brain interactions. Cardiovascular and mental health are tightly interconnected through shared neurohumoral and inflammatory pathways and common risk factors, including hypertension, diabetes, dyslipidemia, and metabolic dysfunction. Acute stressors, such as psychological stress increase sympathetic tone and hypothalamic–pituitary–adrenal axis activity. This response promotes catecholamine release, systemic inflammation, endothelial dysfunction, and atherosclerosis, thereby linking cardiac and cerebral pathology [108, 109]. Cardiovascular conditions such as heart failure, atrial fibrillation, and coronary artery disease elevate the risk of stroke, cognitive decline, and dementia [110–112], driven by shared mechanisms such as chronic inflammation, autonomic dysregulation, and impaired cerebral perfusion [113]. These heart–brain interactions are further influenced by sex-specific differences in renin–angiotensin–aldosterone system activity, immune signaling, and cerebrovascular regulation [114]. Women more frequently experience depression and anxiety following cardiac events [115], whereas men more often present with earlier-onset coronary disease and distinct autonomic profiles [116].

Overall, accumulating evidence supports CKM syndrome as an integrative framework linking metabolic, kidney, cardiovascular, and brain health. Although direct studies on the full syndrome remain limited, substantial data from its individual components demonstrate shared inflammatory, vascular, neurohumoral, and sex-specific mechanisms that contribute to cognitive decline and dementia risk. These insights highlight the importance of early, sex-informed risk stratification and targeted prevention strategies to mitigate CKM syndrome-related brain complications across the lifespan.

## Conclusion

The molecular mechanisms driving sex- and gender-specific CKM syndrome progression remain incompletely understood, particularly the pathophysiological transition from subclinical to overt CVD. The impact of exogenous hormones and reproductive hormonal shifts, such as menopause in women and age-related testosterone decline in men, on CKM stage progression warrants further investigation and clearer delineation. Compounding these knowledge gaps, current clinical guidelines for obesity, diabetes, and CKD management lack sex- or gender-specific recommendations. Given these uncertainties, clinicians should recognize that sex- or gender-specific risk-enhancing factors, including early menopause, adverse pregnancy outcomes, and polycystic ovary syndrome, warrant earlier CKM syndrome screening and more aggressive lifestyle intervention.

## Data Availability

No datasets were generated or analysed during the current study.

## Abbreviations

AD: Alzheimer’s disease
BMI: Body mass index
CKD: Chronic kidney disease
CKM: Cardio–kidney–metabolic
CVD: Cardiovascular disease
ERα: Estrogen receptor–α
FDA: Food and Drug Administration
HDL: High–density lipoprotein
IR: Insulin resistance
LDL: Low–density lipoprotein
MHT: Menopausal hormone therapy
NHANES: National Health and Nutrition Examination Survey
PCOS: Polycystic ovary syndrome
T2D: Type 2 diabetes
VAT: Visceral adipose tissue
